# Genomic Landscape of Non-Small Cell Lung Cancer (NSCLC) in East Asia Using Circulating Tumor DNA (ctDNA) in Clinical Practice

**DOI:** 10.3390/curroncol29030174

**Published:** 2022-03-21

**Authors:** Byoung Chul Cho, Herbert H. F. Loong, Chun-Ming Tsai, Man Lung P. Teo, Hye Ryun Kim, Sun Min Lim, Suyog Jain, Steve Olsen, Keunchil Park

**Affiliations:** 1 Division of Medical Oncology, Yonsei Cancer Center, Seoul 03722, Korea; cbc1971@yuhs.ac (B.C.C.); nobelg@yuhs.ac (H.R.K.); limlove2008@yuhs.ac (S.M.L.); 2 Department of Clinical Oncology, The Chinese University of Hong Kong, Hong Kong SAR, China; h_loong@clo.cuhk.edu.hk; 3 Department of Oncology, Veterans General Hospital, Taipei 112, Taiwan; doc3006a@gmail.com; 4 ICON Cancer Centre, Hong Kong SAR, China; icc.central1033@icon.team; 5 Department of Medical Affairs, Guardant Health AMEA, Singapore 138543, Singapore; solsen@guardantamea.com; 6 Sungkyunkwan University School of Medicine, Seoul 2066, Korea; kpark@skku.edu

**Keywords:** genomic profiling, next-generation sequencing, liquid biopsy, non-small cell lung cancer, East Asia

## Abstract

Plasma-based next-generation sequencing (NGS) has demonstrated the potential to guide the personalized treatment of non-small cell lung cancer (NSCLC). Inherent differences in mutational genomic profiles of NSCLC exist between Asian and Western populations. However, the published mutational genomic data of NSCLC has largely focused on Western populations. We retrospectively analyzed results from comprehensive NGS of plasma (Guardant360^®^) from patients with advanced non-squamous NSCLC, as seen in clinical practice. Tests were ordered between January 2016 and December 2020 in Hong Kong, Korea, Taiwan, Japan and Southeast Asia. The assay identified single-nucleotide variants (SNV), insertions and deletions, and fusions and amplifications in 74 genes. In total, 1608 plasma samples from patients with advanced non-squamous NSCLC were tested. The median turnaround time for test results was 7 days. Of the samples with detectable ctDNA (85.6%), 68.3% had alterations in at least one NCCN-recommended NSCLC biomarker. EGFR driver mutations were most frequent (48.6%), followed by alterations of KRAS (7.9%), ERBB2 (4.1%) and ALK (2.5%). Co-mutations of EGFR and KRAS occurred in 4.7% of samples. KRAS G12C was identified in 18.6% of all samples with KRAS mutations. Common mutations, such as exon 19 deletions and L858R, accounted for 88.4% of EGFR driver mutations. Among the samples with any EGFR driver mutation, T790M was present in 36.9%, including 7.7% with additional alterations associated with osimertinib resistance (MET amplification, C797X). Comprehensive plasma-based NGS provided the timely and clinically informative mutational genomic profiling of advanced non-squamous NSCLC in East Asian patients.

## 1. Introduction

Lung cancer—of which non-small cell lung cancer (NSCLC) comprises the majority of cases—is the second-most frequently diagnosed cancer worldwide [1]. With advances in precision oncology and targeted therapies, the timely, comprehensive, and accurate identification of potentially actionable genomic alterations is crucial for improving patient outcomes. Within NSCLC, the effective and comprehensive characterization of individual drivers and resistance mutations in key biomarkers, including *EGFR* and *ALK*, holds the key to the personalization of NSCLC treatment selection and monitoring [2]. For advanced non-squamous NSCLC, international treatment guidelines currently recommend testing for genomic alterations in more than 10 different genes [3]. Even when a non-actionable mutation in a driver gene is detected, such as in the majority of KRAS mutations, the results of genomic testing are considered informative because such a finding indicates that an actionable driver mutation in another gene is unlikely [2].

Tumor tissue has traditionally been the standard material from which to identify the presence of potentially actionable biomarkers [2,4], with guidelines recommending tissue-based genomic or protein-based testing at the initial diagnosis of metastatic NSCLC [5,6,7,8,9]. However, there are challenges associated with tissue biopsies. Sufficient tissue for genomic profiling is unavailable for 20–30% of patients [4,6] Additionally, in cases of intratumor and intertumor heterogeneity, a single biopsy specimen may not be fully representative of a tumor’s global mutational profile [10,11,12].

Given its minimal invasiveness compared with tissue biopsies [13], liquid biopsy has been employed in a range of clinical scenarios to assess genomic alterations in advanced cancers [2,4]. NSCLC guidelines have recognized liquid biopsies as valid alternatives to tissue biopsies in cases where tumor tissue is limited or unavailable, particularly when invasive tissue sampling poses an unacceptable risk or burden [5,8,9,14]. The International Association for the Study of Lung Cancer (IASLC) recommends liquid biopsy as one of the preferred methods for genotyping newly diagnosed and after-progression patients with advanced NSCLC [9]. Additionally, the American Society of Clinical Oncology guidelines advocate that the safety, speed and convenience of liquid biopsies may even be of value for patients ordinarily able to undergo tissue biopsy [14].

Liquid-biopsy-based NGS allows for a comprehensive characterization of the genomic landscape of cell-free DNA in cancer patients [2,4]. Repeated testing and analysis of cell-free DNA enable the monitoring of the evolution of clonal variations and resistance and strategic treatment selection throughout the course of treatment [2,4]. The National Comprehensive Cancer Network (NCCN) cites evidence that cell-free DNA may be used to identify actionable mutations that would otherwise not be identified [5], while the College of American Pathologists/IASLC/Association for Molecular Pathology guidelines suggest that cell-free DNA analysis may be preferred over tissue biopsy for the detection of *EGFR* T790M at progression for a comprehensive assessment of genomic and subclonal alterations [8]. Most guidelines still mention the need for tissue biopsy in cases with a negative liquid biopsy test [9]. The Guardant360^®^ assay (Guardant Health Inc; Redwood City, CA, USA) is the first US Food and Drug Administration (FDA)-approved companion diagnostic test that utilizes plasma-based NGS technology [7,15]. This assay assesses ctDNA for 74 genes from a single blood test within seven days of sample receipt in the laboratory, preventing the need for multiple single-gene hotspot tests. The assay is indicated for patients with advanced solid tumors at diagnosis and progression. Validation studies of this ctDNA-based NGS assay have shown that discovery rates for actionable alterations using plasma-based genotyping are similar to tissue-based genotyping, with a high sensitivity, specificity, and concordance [11,15]. 

The evidence basis for the clinical utility of plasma-based genotyping is still developing. Published genomic landscape data for NSCLC are derived primarily from studies in Western populations [16]; smaller cohorts from Asia have been reported [17]. The proportion of EGFR mutations is much higher in patients with non-squamous NSCLC in Asia (30–40%) than those in the US and Europe (10–15%) [18,19,20]. On the other hand, KRAS mutations are less common in East Asian patients with NSCLC (8–10%) than in Western patients (26%) [21]. Here, we describe the landscape of mutational alterations in East Asian patients with advanced non-squamous NSCLC, whose plasma samples were analyzed with a commercially available, ctDNA-based NGS assay as part of standard clinical practice.

## 2. Materials and Methods

### 2.1. Study Design

We analyzed Guardant360 results from tests ordered between January 2016 and December 2020 in Hong Kong, Korea, Taiwan, Japan and Southeast Asia. Whole blood samples from patients with advanced non-squamous NSCLC were obtained and included in the analysis. Samples designated as “non-small cell lung cancer” without additional details were included. We excluded samples collected from patients with neuroendocrine (including small cell) carcinoma, sarcomatoid, or pure squamous NSCLC, and from patients enrolled in prospective clinical trials. 

### 2.2. Cell-Free Next Generation Sequencing

The Guardant360 assay is a comprehensive genomic profiling assay that identifies single-nucleotide variants (SNVs), insertions and deletions, fusions, and amplifications [15]. The assay covers complete exon sequencing of multiple genes, including EGFR, ERBB2, and KRAS. During the collection period, the assay included 70 to 74 genes (v2.9 to v2.11). All samples were analyzed by a single Clinical Laboratory Improvement Amendments (CLIA)-certified and CAP-accredited laboratory in California. 

In the present analysis, clinically relevant and guideline-recommended biomarkers included mutations in EGFR, ERBB2, and KRAS; BRAF V600E; MET amplification and exon 14 skipping; and ALK, ROS1, RET, or NTRK1 fusions. Synonymous mutations and variants of unknown significance were not considered to be clinically relevant but were included as indicators of tumor DNA in the plasma. 

### 2.3. Statistical Analyses

Descriptive statistics were used to report sample characteristics and mutation types and frequencies. In samples with multiple concurrent potential driver alterations, the following conventions were applied to categorize the primary driver. In most cases, the alteration with a higher variant allelic frequency (VAF) was considered as the primary driver. In samples with MET amplification and either EGFR mutation or ALK fusion, the EGFR mutation or ALK fusion was classified as the primary driver. Samples with potential driver mutations in separate genes with similar or relatable VAFs were classified as compound mutations.

## 3. Results

### 3.1. Sample Characteristics

**Figure 1:**
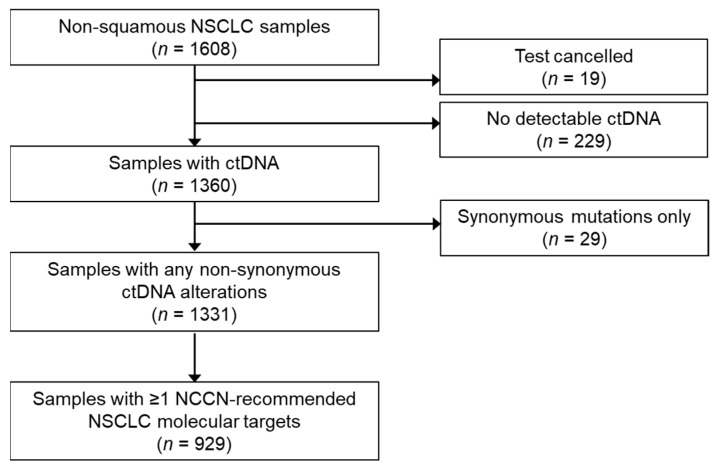
Characteristics of non-squamous NSCLC samples tested. Abbreviations: ctDNA, circulating tumor DNA; NCCN, National Comprehensive Cancer Network; NSCLC, non-small cell lung cancer.

A total of 1608 plasma samples from patients with advanced non-squamous NSCLC were tested (Figure 1). The samples were from 810 women and 641 men; patients had a median age of 62 (range 28–94) years. The median turnaround time from the sample receipt in the laboratory to reporting was seven days. The median turnaround time from the blood draw to results was nine days. ctDNA was identified in 1360 tested samples (detection rate 85.6%), with a median VAF of 0.6%. 

### 3.2. Detection of Driver Mutations

**Figure 2:**
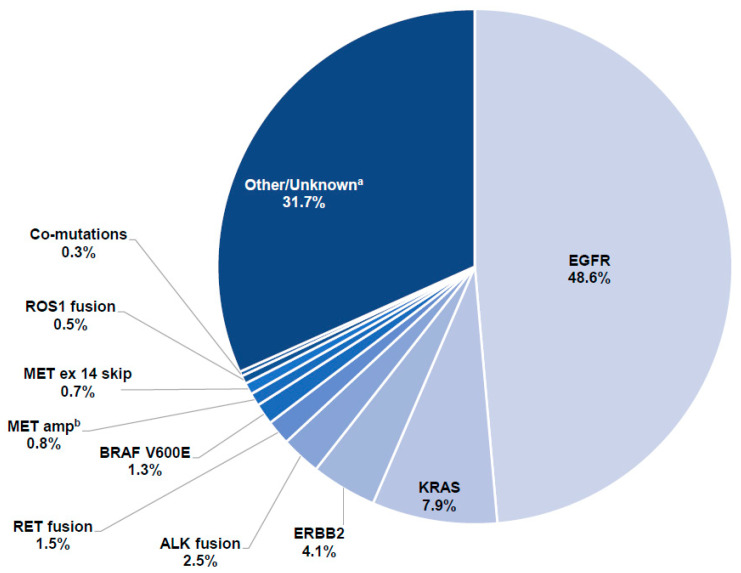
Driver mutations identified in 1360 samples with detectable ctDNA. ^a^ Any alteration other than the listed driver alterations; ^b^ Without any other potential driver alterations. Abbreviations: amp, amplification; ctDNA, circulating tumor DNA; ex, exon; skip, skipping.

The driver mutations identified in 1360 samples with detectable ctDNA are described in Figure 2. Two-thirds of the samples (*n* = 929; 68.3%) had alterations in—≥1 NCCN-recommended NSCLC molecular target [3]. *EGFR* was the most frequently mutated driver gene (48.6%), followed by *KRAS* (7.9%), *ERBB2* (4.1%) and *ALK* (2.5%). Concurrent *EGFR* and *KRAS* mutations were identified in 4.7% of samples. *KRAS* G12C was identified in 18.6% of all samples with *KRAS* mutations (Supplementary Figure S1). 

### 3.3. EGFR Mutations

*EGFR* mutations were the most common driver mutation in the study cohort and included cases with single as well as multiple mutations in the gene. The samples were classified by the presence of various *EGFR* driver mutations, and then reclassified by the presence of *EGFR* resistance mutations.

#### 3.3.1. Classification of Samples with EGFR Alterations by Driver Mutation

**Figure 3:**
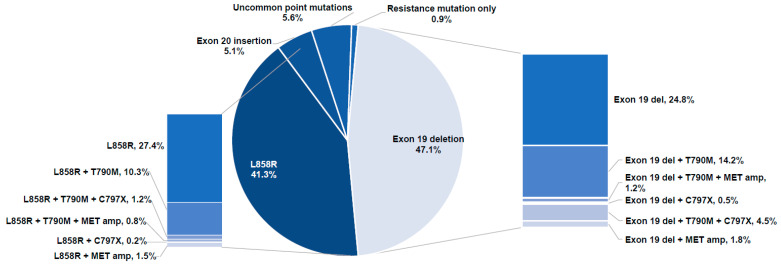
Classification of samples with EGFR driver mutations with a breakdown of L858R mutations (**left**) and exon 19 deletions (**right**). Abbreviations: amp, amplification; del, deletion.

**Figure 4:**
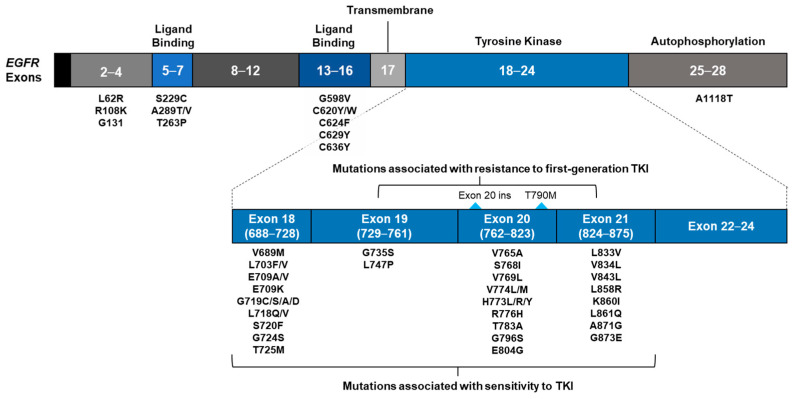
Potentially actionable alterations of the EGFR gene detected in ctDNA in this study. Abbreviations: ctDNA, circulating tumor DNA; EGFR, epidermal growth factor receptor; del, deletion; ins, insertion; SNV, single-nucleotide variant; TKI, tyrosine kinase inhibitor.

The 661 samples with *EGFR* driver mutations are classified in Figure 3 to depict the proportion of *EGFR* driver mutations. The majority of samples (88.4%) contained the common *EGFR* driver mutations, such as exon 19 deletions (47.1%) and L858R (41.3%). Other potentially actionable driver mutations included exon 20 insertions (5.1%) and uncommon point mutations (5.6%). Examples of uncommon mutations represented in two or more samples included G719X, L861Q, E709X, H773X, L718X, E709A (Supplementary Table S1). A complete list of potentially actionable *EGFR* mutations, as found in this database, is provided in Figure 4. 

#### 3.3.2. Classification of Samples with EGFR Mutations by Presence of EGFR TKI Resistance Alterations

Of 661 samples with identified *EGFR* mutations, most (62.2%) had driver mutations without co-occurring resistance alterations (Table 1). T790M was the most common resistance mutation identified in these samples (33.6%). It constituted the only known resistance mutation in 25.0% of samples but was found in conjunction with additional resistance alterations in 7.7%. Most of these additional alterations were generally associated with resistance to osimertinib (*MET* amplification, C797X). Other combinations of *EGFR* driver and resistance mutations were less common.

## 4. Discussion

Based on the results of a commercially available comprehensive plasma-based NGS assay in 1608 samples obtained as part of routine clinical practice from East Asian patients. Over two-thirds (68.3%) of samples with ctDNA had clinically informative alterations. This percentage was higher than that reported for the same assay in prospective studies conducted in Western countries (27–29%) [7,22]. This is largely driven by the prevalence of EGFR mutations in Asian NSCLC patients.

Nearly half (48.6%) of all blood samples had *EGFR* mutations, similar to reports of tissue samples from Asia; another 19.7% had other informative alterations (e.g., *KRAS, ERBB2*, and *ALK*). These findings highlight the need to test for a wide range of genomic alterations to discover potentially actionable alterations and enable personalized treatment. With the approach used here, all relevant information could be obtained from a single blood draw in under 10 days from collection. 

Despite calls for broader testing, genomic testing rates for patients with advanced stage NSCLC remain relatively low [23]. Real-world studies demonstrated that less than 10% of patients with advanced NSCLC received testing for all NCCN-recommended biomarkers [7,24]. Biomarker testing rates in patients with NSCLC in East Asia vary according to region, for example, 43% of patients in North China compared with 83% of patients in Japan [25,26]. The ctDNA detection rate in our analysis is 85.6%, which is consistent with the 89.8% reported with the same assay in predominantly Western patients in a similar setting [27]. When applied to clinical studies that limit testing to patients with confirmed advanced stage NSLC, the ctDNA detection rate of the assay is even higher at 95% [6]. The frequencies of specific genomic alterations are similar to reports of tissue analysis for untreated non-squamous NSCLC in Asia [28]. However, our results do not necessarily reflect the true prevalence of specific alterations, as the selection of patients who were tested was not controlled. In some cases, plasma-based NGS may have been considered after the failure of standard techniques used to detect common mutations (*EGFR*, *ALK*), which may have reduced the observed frequency of certain driver mutations in the study population. On the other hand, for patients who experienced disease progression after targeted therapy for a known driver, physicians may have preferred a blood-based assay over a repeat tissue biopsy. This could have enriched our clinical use population for samples from patients whose tumors had established NSCLC driver mutations. 

An improved understanding of the mutational landscape of NSCLC has enabled the development of mutation-specific therapies. Effective treatment has been established for many uncommon *EGFR*-activating mutations [29], and there has been much effort to develop medicines that target *EGFR* exon 20 insertions [30,31] and C797X mutations [32]. Unlike *EGFR* hotspot testing, a complete exonic analysis of the *EGFR* gene by the NGS of tumor-derived materials (ctDNA or tissue) can facilitate a thorough assessment of *EGFR* mutations to guide patients toward the most appropriate treatment. 

Our findings revealed that 36.9% of samples had *EGFR* driver mutations co-occurring with resistance mutations, of which T790M was the most common. The ability of ctDNA-based NGS to identify both resistance mutations and driver mutations within a sample makes it useful not only for identifying treatment history but also for studying the possible mechanisms of acquired resistance to *EGFR* TKIs [33], potentially guiding informed treatment choices at successive lines of therapy. The predominance of *EGFR* mutations observed in our study is also consistent with that reported by studies utilizing ctDNA or tissue for genotyping in Asian populations [28,34,35]. Taken together, these findings suggest that the ctDNA-based NGS assay has comparable diagnostic accuracy to tissue-based genotyping. 

In this analysis, the ctDNA-based assay also identified uncommon *EGFR* mutations, including G719X, E709A and other SNVs, in 5.6% of the samples. Of these, uncommon, yet potentially actionable, *EGFR* mutations (2.7%) were more prevalent than actionable biomarkers in other genes. 

In addition to *EGFR* mutations, other key guideline-recommended genomic biomarkers were detected in 19.7% of patients. *KRAS* mutations were found in 7.9% of the samples, which is similar to other Asian studies [36]. *KRAS* G12C—a potentially actionable mutation targetable by emerging TKIs [37]—contributed to 18.6% of all *KRAS* mutations in this study and is lower than the 33% reported in another Asian study [38]. Driver mutations in NSCLC are generally considered to be mutually exclusive; however, the co-occurrence of *KRAS* and *EGFR* mutations has been reported [39]. In our study, 4.7% of samples with *KRAS* driver mutations co-occurred with *EGFR* mutations; this finding is consistent with the 4.3% reported in a recent real-world study of Asian patients with NSCLC [40]. 

The median VAF reported in this analysis was 0.6%, which is similar to that reported in larger analyses of plasma from patients with a broad range of tumor types tested with the same assay [11]. Clinical studies have demonstrated that the efficacy of targeted therapy is independent of relevant mutational VAF in ctDNA [22,41]. Due to variations in tumor DNA shedding, concentrations of ctDNA may be very low in plasma. Therefore, the consideration of ctDNA NGS test sensitivity should be a factor when selecting which test to order. 

The ctDNA-based NGS assay used in this study is performed by a high-volume, CAP-accredited, and CAP-certified central laboratory as its sole source, thus minimizing the analytical variation that can arise when testing is conducted by local or low-volume laboratories. Our findings provide further real-world evidence that the assay is able to identify a large range of guideline-recommended biomarkers, with analysis results that are consistent with the existing literature.

There are some limitations of ctDNA-based NGS technology. The concentration of ctDNA in plasma is dependent on total tumor DNA shedding, which may be limited in cases of lower total body tumor burden or when tumors are located in the central nervous system. In cases of certain complex genomic alterations, such as fusions or large insertions or deletions, sensitivity may be decreased compared to that seen with SNVs. Furthermore, while consideration should be given to non-tumor mutations occurring through clonal hematopoiesis of indeterminate potential (CHIP) [9], actionable NSCLC driver mutations are not among the common alterations associated with CHIP [42]. 

This analysis has several limitations. The clinical history of the patients is unknown. Whether or not the patients had received prior systemic treatment—and which type of treatment—would likely impact the type and frequency of alterations detected. Furthermore, we did not have a testing history of the patients. For some patients, ctDNA NGS testing may have been their first and only analysis. For others, it could have been used as a reflex test following a negative result from another test at the time of disease progression on initial treatment or after failure of multiple lines of therapy. Therefore, the frequency of alterations observed in our study may not be representative of East Asian patients with NSCLC in general. Nevertheless, our findings on the frequency of common driver mutations, especially *EGFR*, are largely consistent with other Asian studies [34,35], and the patients included reflect the real-world experience of the incorporation of plasma-based NGS into the NSCLC diagnostic paradigm.

## 5. Conclusions

Comprehensive plasma-based NGS, as applied in clinical practice, identified relevant biomarkers in patients from East Asia with advanced non-squamous NSCLC. The use of a single assay, which requires only whole blood samples as starting material, identified both common and rare clinically informative mutations in a timely manner, avoiding the need for multiple tests to identify less-common actionable alterations. Our findings demonstrate that plasma-based NGS can be integrated into the diagnostic paradigm for NSCLC in East Asia.

## Figures and Tables

**Table 1:** Classification of samples with EGFR mutations by presence of resistance mutations.

Category	Total, *n* (%) (*n* = 661)
**Driver mutation only**	411 (62.2)
Exon 19 deletion	
L858R	
Exon 20 insertion	
G719X	
Exon 19 insertion	
1 uncommon SNV	
**Driver mutation + T790M**	165 (25.0)
Exon 19 deletion + T790M	
L858R + T790M	
G719X + E709A + T790M	
**Driver mutation + T790M + additional resistance mutation**	51 (7.7)
Exon 19 deletion + T790M + MET amplification	
Exon 19 deletion + T790M + C797X	
L858R + T790M + C797X	
L858R + T790M + MET amplification	
**Driver mutation + T790M + other alterations**	0
N/A	
**Driver mutation + resistance mutation other than T790M**	28 (4.2)
Exon 19 deletion + C797X	
Exon 19 deletion + MET amplification	
L858R + C797X	
L858R + MET amplification	
Exon 20 insertion + MET amplification	
1 uncommon SNV + C797X	
**Resistance mutation only**	6 (0.9)
T790M alone	

Abbreviations: N/A, not applicable; SNV, single-nucleotide variant.

## Data Availability

Restrictions apply to the availability of these data. Data were obtained from Guardant Health AMEA and are available from the authors with the permission of Guardant Health AMEA.

## Supplementary Materials

The following supporting information can be downloaded at: https://www.mdpi.com/article/10.3390/curroncol29030174/s1, Figure S1: Classification of KRAS mutations, Table S1: List of uncommon single-nucleotide variants.
