# Supplementary material for: Genomic Landscape of Non-Small Cell Lung Cancer (NSCLC) in East Asia Using Circulating Tumor DNA (ctDNA) in Clinical Practice
Source: Curr Oncol. 2022 Mar 21;29(3):2154–64. doi: 10.3390/curroncol29030174 (PMC8946965; doi:10.3390/curroncol29030174)
Supplement: Supplementary file 1 [file curroncol-29-00174-s001.zip › curroncol-1593954-supplementary.pdf]

Current Oncology

Supplementary Material

Genomic Landscape of Non-Small Cell Lung Cancer (NSCLC) in East Asia Using Circulating Tumor DNA (ctDNA) in Clinical Practice

Byoung Chul Cho [1], Herbert HF Loong [2], Chun-Ming Tsai [3], Man Lung P Teo [4], Hye Ryun Kim [1], Sun Min Lim [1], Suyog Jain [5,*], Steve Olsen [5] and Keunchil Park [6]

[1] Division of Medical Oncology, Yonsei Cancer Center, Seoul 03722, Korea; cbc1971@yuhs.ac (B.C.C.); nobelg@yuhs.ac (H.R.K.); limlove2008@yuhs.ac (S.M.L.)
[2] Department of Clinical Oncology, The Chinese University of Hong Kong, Hong Kong SAR, China; h_loong@clo.cuhk.edu.hk
[3] Department of Oncology, Veterans General Hospital, Taipei 112, Taiwan; doc3006a@gmail.com
[4] ICON Cancer Centre, Hong Kong SAR, China; icc.central1033@icon.team
[5] Department of Medical Affairs, Guardant Health AMEA, Singapore 138543, Singapore; solsen@guardantamea.com
[6] Sungkyunkwan University School of Medicine, Seoul 2066, Korea; kpark@skku.edu
* Correspondence: sjain@guardantamea.com; Tel.: +65-9088-5313

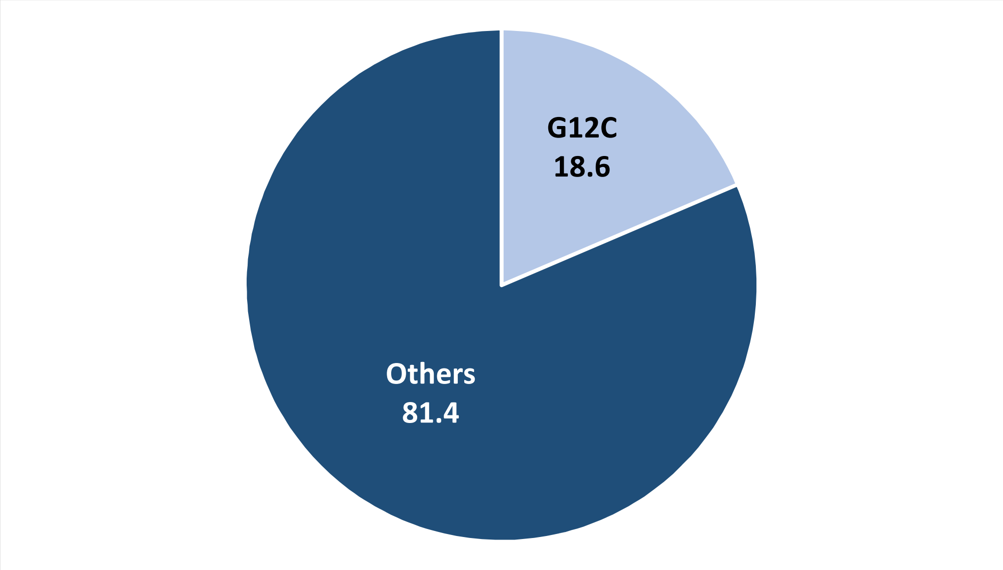

Figure S1. Classification of KRAS mutations.

Table S1. List of uncommon single-nucleotide variants.

EGFR Exon	Alteration
**Exon 2–4**	L62R
	R108K
**Exon 5–7**	S229C
	A289T
	V660A
**Exon 18**	L703V
	E709X (A/K/V)
	L718X (Q/V)
	G719X (A/C/D/S)
	S720F
	G724S
	T725M
**Exon 19**	L747P
**Exon 20**	S768I
	V769L
	H773X (L/R/Y)
	V774M
	R776H
	S811F
**Exon 21**	L833V
	V834L
	L838V
	V843L
	K860I
	L861Q
	A871G
	G873E
**Exon 25–28**	A1118T
	R1199K
